# Poor health status, inappropriate glucose-lowering therapy and high one-year mortality in geriatric patients with type 2 diabetes

**DOI:** 10.1186/s12877-020-01780-9

**Published:** 2020-09-24

**Authors:** Antoine Christiaens, Benoit Boland, Marie Germanidis, Olivia Dalleur, Séverine Henrard

**Affiliations:** 1 Fund for Scientific Research – FNRS, Brussels, Belgium; 2 Clinical Pharmacy Research Group, Louvain Drug Research Institute (LDRI), Université catholique de Louvain, Brussels, Belgium; 3 Institute of Health and Society (IRSS), Université catholique de Louvain, 30, Clos Chapelle-Aux-Champs bte B1.30.15, 1200 Brussels, Belgium; 4 Geriatric Medicine Unit, Cliniques Universitaires Saint-Luc, Université catholique de Louvain, Brussels, Belgium; 5 Pharmacy Department, Cliniques Universitaires Saint-Luc, Université Catholique de Louvain, Brussels, Belgium

**Keywords:** Geriatric patients, Health status, Mortality, Type 2 diabetes, Glucose-lowering therapy, Overtreatment

## Abstract

**Background:**

Glucose-lowering therapy (GLT) should be individualized in older patients with type 2 diabetes (T2D) according to their health status and their life expectancy. This study aimed at assessing the inappropriateness of GLT prescribing and the one-year mortality rate in geriatric patients with T2D.

**Methods:**

Retrospective cohort study of consecutive inpatients with T2D admitted to a geriatric ward of a Belgian university hospital. Inclusion criteria were age ≥ 75 years, T2D with GLT before admission, and HbA1c measurement during the hospital stay. Comorbidities and geriatric syndromes were collected. GLT agents were classified into hypoglycaemic and non-hypoglycaemic ones, and their dosages were expressed in daily defined dose (DDD). Health status (intermediate or poor) and GLT appropriateness (appropriate, overtreatment, undertreatment) were assessed according to the 2019 Endocrine Society guideline on diabetes treatment in older adults, in which GLT overtreatment requires the presence of hypoglycaemic therapy. One-year mortality was determined using the National Registry of vital status, and its associated factors were analysed using multivariable Cox’ regression.

**Results:**

The 318 geriatric patients with T2D (median age 84 years; 46% female) were in intermediate (33%) or poor health (67%). These two groups reached similar low HbA1c values (median 6.9%) with similar GLT regimens. GLT overtreatment was frequent (57%) irrespectively of the geriatric features. One-year mortality rate was high (38.5%) and associated in multivariate analysis with poor health status (HR: 1.59, *p* = 0.033), malnutrition (HR: 1.67, *p* = 0.006) and GLT overtreatment (HR: 1.73, *p* = 0.023). Patients with GLT overtreatment had a higher mortality rate (44.5%).

**Conclusions:**

GLT overtreatment was present in more than half of these geriatric patients. Many of them were in poor health status and died within one-year. Special attention should be paid to individualisation of the HbA1c goals in the geriatric patients with diabetes, and to GLT de-intensification in those being over-treated.

## Background

In older people, those with a geriatric profile are among the frailest, the most dependent and those with the shortest life expectancy. In this population, type 2 diabetes (T2D) is prevalent [1] and the associated glucose-lowering therapy (GLT) can be complex to manage. The treatment should be moderate enough to avoid as possible hypoglycaemic events while remaining intense enough to control high-level hyperglycaemia related symptoms. In older patients with geriatric features, i.e. those with frail profile and complex or poor health status, the hypoglycaemic events are indeed particularly harmful as they increase risk of falls, falls-related fractures, coma, seizures and cognitive impairments as well as all causes mortality [2]. These geriatric patients are at higher risk of more frequent and severe hypoglycaemic events [2–5].

In recent years, Scientific Societies and expert panels published clinical practice guidelines addressing the need to individualise GLT in older patients with T2D in order to minimize the risk of GLT-associated hypoglycaemia. They recommended several HbA1c target ranges according to the patient’s health status (American Geriatric Society 2013 [6], American Diabetes Association 2020 [7]), life expectancy (European Association for the Study of Diabetes 2015 [8]) or geriatric profile (frailty, dementia: International Diabetes Federation 2013 [9]). In 2019, the Endocrine Society (co-sponsored by the European Society of Endocrinology, The Gerontological Society of America, and The Obesity Society) released a clinical practice guideline for the treatment of diabetes in older people [10]. This guideline helps operationalising the tailoring of the HbA1c target range based on the patient’s health status (good, intermediate or poor) and GLT regimen (presence of a hypoglycaemic agent or not). In this guideline, HbA1c should not be lower than 7, 7.5 and 8% in patients on hypoglycaemic medications with, respectively, good, intermediate and poor health status. Patients on hypoglycaemic medications with HbA1c values lower than these cut-off values may therefore be considered as over-treated.

The aims of the present study was to assess (a) the prevalence of GLT inappropriate prescribing in geriatric patients with type 2 diabetes according to the 2019 Endocrine Society guideline [10] and (b) the one-year mortality rate and its associated factors.

## Methods

### Study design and participants’ inclusion

This retrospective study included all consecutive inpatients with type 2 diabetes admitted in a geriatric ward (24 beds) of an academic hospital (Brussels, Belgium) between 2008 and 2015. Inclusion criteria were age ≥ 75 years, type 2 diabetes, glucose lowering therapy (GLT) at home, and HbA1c measurement during the hospital stay. In patients with multiple hospital stay, only the first one was considered in this study.

### Data collection and definition of variables

Data was extracted from the patient’s medical record and included general, geriatric and biomedical characteristics, as well as information about GLT at home. Type 2 diabetes was defined according to the Expert Committee on the Diagnosis and Classification of Diabetes Mellitus [11]. Geriatric characteristics included residence in a long-term nursing facility, chronic functional impairment defined by ≥2 impairments in 5 of the basic activities of daily living (i.e. eating, bathing, dressing, toileting and transferring) [10], malnutrition (diagnosed by a full-time dietician after taking an anamnesis about patient’s appetite loss, weight loss, eating habits as their evolution over time), recent falls (≥ 2 falls within last year) and chronic cognitive impairment (dementia or MMSE < 24/30). Estimated glomerular filtration rate (eGFR) was computed using MDRD formula [12] based on the creatinine rate at the admission in geriatric ward; eGFR < 30 ml.min^− 1^ defined severe renal failure. Glycated haemoglobin (HbA1c) was expressed in NGSP nomenclature (%). GLT agents were encoded according to the Anatomical Therapeutic Chemical (ATC) classification system [13]. Hypoglycaemic agents included insulins (A10A), sulfonylureas (A10BB) and glinides (A10BX02–03–05-08). Non-hypoglycaemic agents included biguanides (A10BA), GLP1-receptor agonists (A10BJ), DPP4-inhibitors (A10BH), alpha-glucosidase inhibitors (A10BF) and thiazolidinediones (A10BG). Doses of each GLT agent were converted into Defined Daily Dose (DDD), according to the ATC/DDD Index 2018 [13]. GLT was considered as intense when the dose was ≥1.0 DDD in this geriatric population. Finally, the patient’s vital status was collected using the Belgian national Register one-year after the hospital admission, this time frame being suited for the study population.

### Overall health status

Patient overall health status was classified according to the 2019 Endocrine Society guideline criteria (comorbidities, functional status, cognitive status and residence) [10] as good (absence of diabetic comorbidities, ≤ 2 non-diabetes chronic illnesses, no basic ADL impairments and ≤ 1 instrumental ADL impairment), intermediate (≥ 3 non-diabetes chronic illnesses, mild cognitive impairment/early dementia or ≥ 2 IADL impairments), or poor (end-stage medical condition, moderate/severe dementia, ≥ 2/5 ADL impairments or residence in a long-term nursing facility).

### Appropriateness of glucose lowering therapy

The 2019 Endocrine Society Guideline [10] defines the patient’s HbA1c target range based on the overall health status and the use of hypoglycaemic therapy (i.e. insulins, sulfonylureas or glinides). In the presence of hypoglycaemic therapy, the HbA1c range has a lower limit. In patients with good health status, the HbA1c target range is < 7.5% in the absence of hypoglycaemic therapy and 7.0–7.5% in the presence of hypoglycaemic therapy. In patients with intermediate health status, the HbA1c target range is < 8.0% and 7.5–8.0%, respectively, in the presence and the absence of hypoglycaemic therapy. In patients with poor health status, the HbA1c target range is < 8.5% and 8.0–8.5% in the presence and the absence of hypoglycaemic therapy.

Participants were classified into one of three categories of GLT appropriateness, i.e. appropriate GLT (HbA1c value in the patient’s target range), GLT undertreatment (HbA1c value higher than the patient’s target range) and GLT overtreatment (HbA1c value lower than the patient’s target range). As target ranges of HbA1c have a lower bound only for people using hypoglycaemic agents, GLT overtreatment concerned only patients receiving hypoglycaemic therapy. Table 1 presented the different cut-offs used to define the three categories of GLT appropriateness based on the suggested HbA1c target ranges.

**Table 1:** Definition of categories of GLT appropriateness (undertreatment, appropriate GLT and overtreatment) according to the concordance with the 2019 Endocrine Society Guideline [10]

Use of hypoglycaemic agents^a^	Overall health status	GLT appropriateness category		
		Appropriate GLT	Undertreatment	Overtreatment
		HbA1c level	HbA1c level	HbA1c level
No	Good	< 7.5%	≥ 7.5%	/
	Intermediate	< 8.0%	≥ 8.0%	/
	Poor	< 8.5%	≥ 8.5%	/
Yes	Good	≥ 7.0 and < 7.5%	≥ 7.5%	< 7.0%
	Intermediate	≥ 7.5 and < 8.0%	≥ 8.0%	< 7.5%
	Poor	≥ 8.0 and < 8.5%	≥ 8.5%	< 8.0%

^a^Hypoglycaemic agents include insulins, sulfonylureas or glinides; *GLT* Glucose-lowering therapy

The terms “appropriate” and “inappropriate” should be understood as “concordant with the guideline” and “non-concordant with the guideline” respectively.

### Statistical analyses

Continuous data were expressed as median [P25; P75] and categorical data as number and percentages. Comparisons between the three GLT appropriateness categories were performed using Kruskal-Wallis test for continuous variables and Pearson’s chi-squared test or Fisher-Freeman-Halton test for categorical variables. Factors associated with GLT appropriateness categories were assessed using a multinomial logistic regression. All variables associated with a *p*-value < 0.2 in univariate analysis were candidate for the multivariable model and a stepwise selection using Akaike information criterion (AIC) was performed to select the final multivariable model. Multicollinearity was assessed using variance inflation factor (VIF), a VIF value > 5 indicating multicollinearity. Factors associated with 1-year mortality were assessed using a Cox’s Proportional Hazards regression. The selection of the final multivariable model was performed in the same way as for the multinomial logistic regression above. Validity conditions were fulfilled, proportional hazards hypothesis was respected and censoring was non-informative. Statistical analyses were performed using R software (version 3.4.1). A *p*-value < 0.05 was considered statistically significant.

### Ethical consideration

This study was approved by the Institutional Review Board Committee (Commission d’Ethique Hospitalo-Facultaire, Cliniques universitaires Saint-Luc, Brussels, Belgium, IRB agreement nb. IRB00001530 and IRB00008535).

## Results

This study included the 318 consecutive patients with T2D admitted to the geriatric ward. According to the 2019 Endocrine Society guidelines, the patient’s overall health was poor (*n* = 213, 67.0%) or intermediate (*n* = 105, 33.0%), no patient with T2D admitted to the geriatric ward being in good health because of some medical comorbidities or/and functional dependencies.

### Patients’ characteristics

The median age was of 84.0 years, 45.9% of the patients were female, and 22.3% lived in a long-term nursing facility (Table 2). Among T2D comorbidities, patients presented ischaemic heart disease (42.8%) and severe renal impairment (16.8%). The median number of daily drugs was 9 (P25-P75: 7–11). Geriatric features were prevalent, namely functional impairment (63.2%), cognitive impairment (57.5%), recent falls (53.1%), severe polypharmacy (48.8%) and malnutrition (30.2%) (Table 2). Patients in poor health (*n* = 213, 67.0%) did not differ from those in intermediate health (*n* = 105, 33.0%) in age, sex, number of comorbidities or daily drugs, neither in features of glucose lowering therapy (GLT), i.e. use of hypoglycaemic agents (81.7% vs. 75.2%; *p* = 0.180), use of metformin (38.5% vs. 46.7%; *p* = 0.164), and overall GLT DDD (0.85 vs. 0.81 DDD; *p* = 0.316).

**Table 2:** Patient’s and glucose-lowering therapy (GLT) characteristics, according to GLT appropriateness (*N* = 318)

Variable	All patients	Appropriate GLT	GLT Overtreatment	GLT Undertreatment	*p*-value
	*n* = 318	*n* = 79 (24.8%)	*n* = 182 (57.2%)	*n* = 57 (17.9%)	
	Median [P25; P75] or n (%)	Median [P25; P75] or n (%)	Median [P25; P75] or n (%)	Median [P25; P75] or n (%)	
Age, in years	84 [80; 88]	84 [80; 87]	84 [81; 88]	83 [80; 87]	0.544
Female	146 (45.9)	34 (43.0)	89 (48.9)	23 (40.4)	0.443
Overall health category					
Intermediate overall health	105 (33.0)	33 (41.8)	52 (28.6)	20 (35.1)	0.107
Poor overall health	213 (67.0)	46 (58.2)	130 (71.4)	37 (64.9)	
Comorbidities					
Ischaemic heart disease	136 (42.8)	29 (36.7)	77 (42.3)	30 (52.6)	0.177
Renal failure (*n* = 303)^a^	51 (16.8)	6 (7.9)	36 (21.1)	9 (16.1)	0.038
Geriatric features					
Nursing home residency	71 (22.3)	18 (22.8)	45 (24.7)	8 (14.0)	0.238
Functional impairment ^b^	201 (63.2)	44 (56.7)	123 (67.6)	34 (59.6)	0.155
Severe polypharmacy ^c^	139 (43.7)	33 (48.8)	82 (45.1)	24 (42.1)	0.855
Cognitive impairment	183 (57.5)	46 (58.2)	106 (58.2)	33 (57.9)	0.999
Recent falls	169 (53.1)	43 (54.4)	93 (51.1)	33 (57.9)	0.646
Malnutrition	96 (30.2)	28 (35.4)	52 (28.6)	16 (28.1)	0.501
GLT characteristics					
HbA1c, in %	6.9 [6.1; 7.8]	6.8 [6.1; 7.6]	6.7 [6.1; 7.2]	9.2 [8.6; 10.1]	< 0.001
Use of GLT classes					
Metformin	131 (41.2)	66 (83.5)	46 (25.3)	19 (33.3)	< 0.001
Other NHGA^d^	9 (2.8)	5 (6.3)	3 (1.6)	1 (1.8)	0.101
Hypoglycaemic agents	253 (79.6)	20 (25.3)	182 (100.0)	51 (89.5)	< 0.001
Bi- or tri-therapy	78 (24.5)	12 (15.2)	49 (26.9)	17 (29.8)	0.076
GLT total intensity, in DDD	0.9 [0.5; 1.4]	0.8 [0.4; 1.0]	0.8 [0.5; 1.3]	1.2 [0.8; 2.0]	0.014
0–0.4 DDD	73 (23.0)	27 (34.2)	40 (22.0)	6 (10.5)	
0.5–0.9 DDD	103 (32.4)	29 (36.7)	58 (31.9)	16 (28.1)	0.002
≥ 1 DDD	142 (44.7)	23 (29.1)	84 (46.2)	35 (61.4)	

*GLT* Glucose-lowering therapy, *HbA1c* Glycated haemoglobin, *DDD* Defined daily dose, *NHGA* Non-hypoglycaemic agents; Hypoglycaemic agents include insulin, sulfonylureas and glinides

^a^defined as estimated glomerular filtration rate < 30 ml/min

^b^defined as ≥2 impairments in basic activities of daily living, including eating, bathing, toileting, transferring and dressing

^c^defined as ≥10 drugs/day

^d^Other non-hypoglycaemic agents were DPP4-inhibitors, thiazolidinediones and alpha-glucosidase inhibitors

### Appropriateness of glucose-lowering therapy

Table 2 compares the patients with appropriate GLT (24.8%), GLT overtreatment (57.2%) and GLT undertreatment (17.9%). These three groups did not statistically differ in socio-demographic characteristics, global health status, prevalence of ischemic heart disease and of geriatric features. Renal failure (eGFR < 30 ml/min) was more present in patients with GLT overtreatment (21.1%) than in patients with GLT undertreatment (16.1%) or appropriate GLT (7.9%) (Table 2; *p* = 0.038).

The three categories of GLT appropriateness differed in HbA1c values, GLT classes and GLT intensity (DDD) (Table 2). Patients with appropriate GLT (*n* = 79) showed a median HbA1c of 6.9%, obtained with a simple GLT regimen (metformin 83.5%; monotherapy 84.8%). They were infrequently on hypoglycaemic agents (25.3%) or on GLT ≥ 1.0 DDD (29.1%). Patients with GLT undertreatment (*n* = 57) had median HbA1c value of 9.2% despite the frequent prescribing of hypoglycaemic agents (89%) and of intense GLT (DDD ≥ 1.0: 61.4%). Patients with GLT overtreatment (*n* = 182) were prescribed a more intense GLT regimen than those with appropriate GLT, with a lower use of metformin use (25.3 vs. 83.5%, *p* < 0.001) and a high use of intense GLT (DDD ≥ 1.0: 46.2 vs. 29.1%, *p* = 0.015). In the logistic regression model comparing GLT overtreatment to appropriate GLT (Additional file 1), GLT overtreatment was associated with severe renal failure (OR [95%CI] = 3.49 [1.38; 8.81]), poor health status (OR 1.96 [1.10; 3.51]) and GLT bi- or tri-therapy (OR 2.41 [1.18; 4.94]).

### Factors associated with one-year mortality

**Fig. 1:**
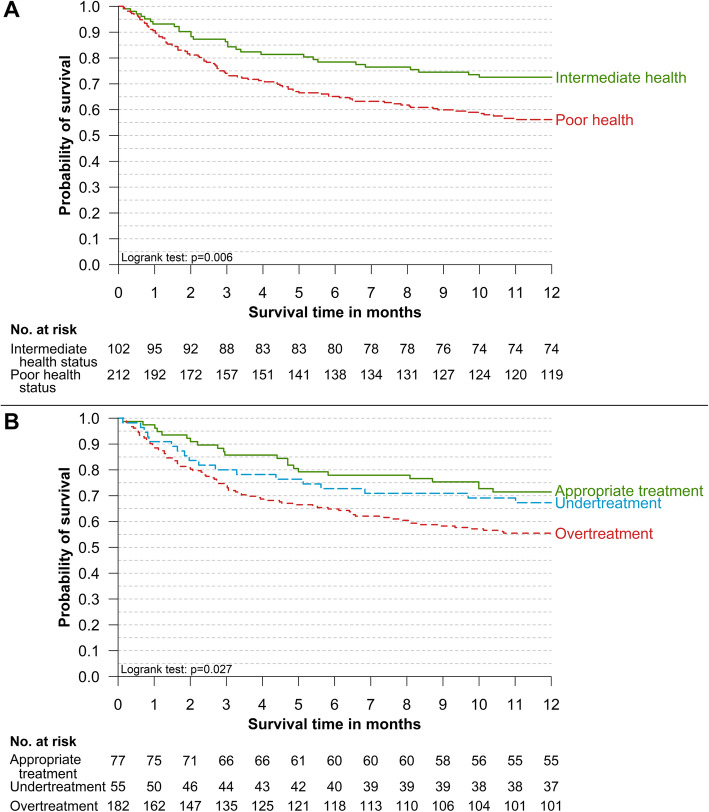
One-year survival of geriatric patients with T2D according to (**a**) Health status and (**b**) GLT-appropriateness. Kaplan-Meier survival curves at 1 year of geriatric patients with type 2 diabetes according to (**a**) their overall health status (Intermediate or Poor) and (**b**) their category of GLT appropriateness (Appropriate, Undertreatment or Overtreatment)

At one year, more than one-third of these geriatric patients with T2D had died (38.5%; *n* = 121/314, 4 missing values). As expected, the one-year mortality rate was higher in patients with poor health status (43.9%) than in those with intermediate health status (27.5%) (Fig. 1a, Logrank test *p* = 0.006). The one-year mortality rate also differed in patients with appropriate GLT (28.6%), GLT undertreatment (32.7%) and GLT overtreatment (44.5%) (Fig. 1b, Logrank test *p* = 0.027). In the multivariable model (Table 3), one-year mortality was not associated with older age, was lower in patients with recent falls (HR: 0.63, *p* = 0.013) and was higher in patients with poor health status (HR: 1.59, *p* = 0.033), malnutrition (HR: 1.67; *p* = 0.006) and GLT overtreatment (vs. appropriate GLT; HR: 1.73, *p* = 0.023). Finally, it was not associated with GLT-undertreatment.

**Table 3:** Factors associated with one-year mortality in Cox Proportional Hazards regression (*N* = 314)

Variables	Univariate model		Multivariable model	
	HR [95% CI]	*p*-value	HR [95% CI]	*p*-value
Socio-demographic characteristics				
Category of ages				
Age < 80 years	1.00			
Age 80–84 years	0.89 [0.53; 1.50]	0.660		
Age 85–89 years	1.04 [0.62; 1.76]	0.879		
Age ≥ 90 years	1.41 [0.80; 2.49]	0.234		
Sex (male vs. female)	1.26 [0.88; 1.80]	0.207		
Health status, comorbidities and geriatric characteristics				
Health status (poor vs. intermediate)	1.80 [1.18; 2.74]	0.007	1.59 [1.04; 2.43]	0.033
Ischaemic heart disease	1.44 [1.01; 2.06]	0.045	1.39 [0.97; 1.98]	0.074
Nursing home residency	1.67 [1.13; 2.48]	0.010		
Malnutrition	1.65 [1.15; 2.38]	0.007	1.67 [1.16; 2.42]	0.006
Recent falls	0.64 [0.45; 0.92]	0.015	0.63 [0.44; 0.91]	0.013
Functional impairment^a^	1.65 [1.11; 2.46]	0.014		
Polypharmacy				
Absent (0–4 drugs/day)	1.00			
Moderate (5–9 drugs/day)	2.08 [0.83; 5.19]	0.117		
Major (≥ 10 drugs/day)	2.41 [0.97; 6.01]	0.059		
GLT characteristics				
Appropriateness				
Present	1.00		1.00	
Undertreatment	1.22 [0.65; 2.27]	0.534	1.22 [0.65; 2.28]	0.542
Overtreatment	1.80 [1.12; 2.89]	0.015	1.73 [1.08; 2.79]	0.023
Category of HbA1c				
HbA1c < 6.5%	1.82 [1.02; 3.22]	0.042		
HbA1c 6.5–7.4%	1.05 [0.58; 1.92]	0.866		
HbA1c 7.5–8.4%	1.00			
HbA1c ≥ 8.5%	1.16 [0.58; 2.33]	0.672		
Use of hypoglycaemic agents	1.71 [1.03; 2.86]	0.040		
Use of metformin	0.62 [0.42; 0.91]	0.015		

*HR* Hazard Ratio, *CI* Confidence interval, *GLT* Glucose-lowering therapy

^a^Functional impairment was defined as ≥2/5 impairments in basic activities of daily living (eating, bathing, dressing, toileting and transferring)

## Discussion

In this study of older old patients admitted to a geriatric ward, GLT at home was appropriately prescribed in only 1 in 4 patients. GLT appropriateness was not associated with any patient’s characteristic but with GLT prescribing, i.e. lower use of hypoglycaemic agents (i.e. insulins, sulfonylureas or glinides) and of intense dose. GLT undertreatment concerned 1 in 6 geriatric patients, in whom HbA1c was too high despite high dose of GLT. GLT overtreatment, i.e. patients prescribed with hypoglycaemic agents with a HbA1c value below the target range, was detected in 1 in 2 patients. GLT overtreatment was associated with poor health status, severe renal failure and use of bi-or tri-therapy of GLT. Importantly, one-year mortality was higher in patients with GLT overtreatment (44%) than those with appropriate GLT, independently of the patient’s health status and of the age of the patient.

GLT overtreatment, which potentially leads to hypoglycaemia [14, 15] and thus to associated comorbidities and mortality [2], was surprisingly not less frequent in patients with geriatric syndromes or poor health status than others. This finding highlights in this population a clear lack of individualisation of GLT according to these characteristics. This is even more surprising since older patients with geriatric features or/and in poor health status are at higher risk of more frequent and severe hypoglycaemic events, due to frequent misdiagnoses, unawareness and atypical presentations [2]. In our study, GLT overtreatment was more frequent in patients with severe renal failure (eGFR< 30 ml/min), most of whom (*n* = 47/51; 92.2%) received at least one hypoglycaemic agent (i.e. insulins, sulfonylureas or glinides). One potential explanation is the contra-indication of metformin in patients with severe renal failure. In addition to the fact that some hypoglycaemic agents can accumulate in case of severe renal failure (e.g. sulfonylureas [16]), other non-hypoglycaemic GLT agents are preferable, such as DPP4-inhibitors, the safety of which (with adjusted doses for some) has been studied in case of severe renal impairment even in older patients [10, 17].

Patients with GLT undertreatment might benefit from GLT intensification in order to avoid discomfort of hyperglycaemia-related symptoms. Beyond the value of HbA1c, the decision to intensify the treatment should be taken with caution. Indeed, hypoglycaemic events can also occur despite high HbA1c values in patients receiving intensive hypoglycaemic therapy [18]. Therefore, in the geriatric patients with a HbA1c over the target level, GLT intensification should be achieved on a case-by-case basis, considering a risk-benefit balance between the discomfort of hyperglycaemia and the risk of hypoglycaemic events. Furthermore, considering that the very old and frail population of this study received highly conservative GLT agents (largely composed by metformin and hypoglycaemic agents), non-hypoglycaemic agents other than metformin could be an interesting option, if further intensification of the treatment is deemed necessary.

The one-year mortality rate was high in these patients (38.5%). In the multivariate model, one-year mortality was higher in the presence of poor health status, low weight and GLT overtreatment, but lower in the presence of multiple falls. The latter association might be explained by the fact that the very dependent geriatric patients do not walk anymore. Falls might indicate a somewhat preserved functional status. The association between one-year mortality and GLT overtreatment is important to discuss. This observation does not mean that GLT overtreatment increases mortality in geriatric patients, as it has been demonstrated in other studies involving younger old and healthier patients [19–21]. Indeed, the observational design of our study does not allow any causal conclusion. Frailty and severe renal failure might be confounding factors, as they are associated to both GLT overtreatment and mortality. However, the observed association between one-year mortality and GLT overtreatment highlights the pointlessness and the risk of intense GLT in geriatric patients with poor health status with a poor one-year life expectancy. It is indeed useless to prescribe an intense GLT therapy with the aim to avoid long-term T2D complications in patients with a short life expectancy, especially since such a therapy induces hypoglycaemic events.

This study was limited by its retrospective design. The duration of diabetes is not known. Data related to the GLT prescribers (e.g. motivations for initiating/continuing this treatment, knowledge about the guidelines on diabetes in older adults) could not be collected. The association of GLT appropriateness with other outcomes that matter to the geriatric patients, i.e. impaired quality of life, hypoglycaemic episodes, functional decline, should be studied in the future. This study was finally limited by its single-centre inclusion, which, despite the risk of selection bias, is to be put into perspective given the continuous inclusion of patients over a long period of time during which several different medical teams succeeded one another.

A strengths of this study is the focus on geriatric patients with type 2 diabetes ≥75 years in the setting of a geriatric ward of a university hospital. Geriatric patients are the most dependent with the most unfavourable health status among older patients (e.g. no patients in this study was in good health status). Therefore, these data cannot be generalised to the general older population ≥ 75 years. However, these data are important for patients from this particular setting, especially as these patients are not commonly represented in the scientific literature on the treatment of type 2 diabetes. Other strengths were the collection of data on the main geriatric syndromes, the tailoring of HbA1c targets according to the 2019 Endocrine Society guideline, and the analysis of the residual life expectancy (vital status at 1 year).

This study confirms the need for an improvement in GLT prescribing in the geriatric patients with T2D. Several actions should be considered. Firstly, the prescribing physician should individualise the HbA1c targets in each older patient based on the health status and the use of hypoglycaemic therapy (i.e. insulins, sulfonylureas or glinides), as suggested by the Endocrine Society. As pointed by most of the recent clinical guidelines on older adults with diabetes, the tailoring of HbA1c is the most effective way to reduce inappropriate therapy and the ensuing risk of hypoglycaemia [10, 22–24]. It is acknowledged that the implementation of guidelines takes time. However, the results of this study highlight the existence and relevance of guidelines related to the individualised management of glucose-lowering therapy, in particular the 2019 Endocrine Society guideline, and to use patients’ health status and the use of hypoglycaemic agents to individualise GLT according the patient’s target HbA1c level. Secondly, the patients should be involved in the decision making process as much as possible [10]. Finally, in the numerous geriatric patients with GLT overtreatment, de-intensification of hypoglycaemic agents (i.e. stopping the medication, reducing the dose or switching to another and safer drug) should be performed especially in patients with a poor overall health status (with frail profile, dementia, cognitive impairment) [25]. Actually, life expectancy of these patients is reduced and the benefit of intensive glucose lowering therapy is therefore absent. Interventional studies are deeply needed to clarify the modalities of GLT de-intensification in older people with type 2 diabetes.

## Conclusions

Inappropriateness of GLT prescribing (i.e. non-concordance with the guideline) was very frequent in these geriatric patients with type 2 diabetes, mainly due to too low HbA1c value with hypoglycaemic agents, i.e. GLT overtreatment. One year-mortality was high and associated with poor health status, low body weight and GLT overtreatment. As the majority of such geriatric patients with diabetes are in poor health and overtreated with GLT, a GLT reassessment should be carried out, in order to improve the appropriateness of GLT prescribing in the geriatric patients with type 2 diabetes.

## Supplementary information

**Additional file 1 **Factors associated with Overtreatment and Undertreatment of GLT (vs. Appropriate-GLT) in multivariable multinomial logistic regression analysis (*n* = 303)

## Data Availability

The datasets generated and/or analysed during the current study are not publicly available due to restrictions on patients’ anonymity but are available from the corresponding author on reasonable request.

## Abbreviations

ADL: Activities of daily living
AIC: Akaike information criteria
ATC: Anatomical Therapeutic Chemical
CI: Confidence interval
DDD: Defined daily dose
GLT: Glucose-lowering therapy
HbA1c: Glycated haemoglobin
HR: Hazard ratio
IADL: Instrumental activities of daily living
MMSE: Mini-mental state examination
T2D: Type 2 diabetes
