# Supplementary material for: Poor health status, inappropriate glucose-lowering therapy and high one-year mortality in geriatric patients with type 2 diabetes
Source: BMC Geriatr. 2020 Sep 24;20:367. doi: 10.1186/s12877-020-01780-9 (PMC7517632; doi:10.1186/s12877-020-01780-9)
Supplement: Supplementary file 1 — Additional file 1 Factors associated with Overtreatment and Undertreatment of GLT (vs. Appropriate-GLT) in multivariable multinomial logistic regression analysis (n = 303) [file 12877_2020_1780_MOESM1_ESM.docx]

**ADDITIONAL FILE 1**

Additional file 1. Factors associated with Overtreatment and Undertreatment of GLT (*vs.* Appropriate-GLT) in multivariable multinomial logistic regression analysis (*n* = 303)

| Variables | Overtreatment *vs.* Appropriate GLT | |  | Undertreatment *vs.* Appropriate GLT | |
| --- | --- | --- | --- | --- | --- |
|  | OR [95% CI] | *p*-value |  | OR [95% CI] | *p*-value |
| Overall health status (poor *vs.* intermediate) | 1.96 [1.10 ; 3.51] | 0.022 |  | 1.34 [0.65 ; 2.76] | 0.420 |
| Renal failure ^a^ | 3.49 [1.38 ; 8.81] | 0.008 |  | 2.51 [0.83 ; 7.60] | 0.103 |
| Bi- or tri-therapy of GLT | 2.41 [1.18 ; 4.94] | 0.016 |  | 2.34 [0.99 ; 5.51] | 0.051 |

OR: Odds Ratio; CI: Confidence interval; GLT: Glucose-lowering therapy; ^a^15 missing values; *defined as estimated glomerular filtration rate <30ml/min.
